# IMPACT OF THE WALK ON! COMMUNITY WALKING PROGRAM ON PSYCHOSOCIAL ATTRIBUTES OF OLDER AFRICAN-AMERICAN WOMEN

**DOI:** 10.1093/geroni/igad104.1696

**Published:** 2023-12-21

**Authors:** Jo Ann Coco-Ripp, Allison Heinrich, Kasual Maxwell-Crudup, Kaliya Hill, Judy Foxworth

**Affiliations:** Winston-Salem State University, Winston-Salem, North Carolina, United States; WFSM, Winston Salem, North Carolina, United States; WSSU, Winston Salem, North Carolina, United States; WSSU, Winston Salem, North Carolina, United States; Winston-Salem State University, Winston-Salem, North Carolina, United States

## Abstract

Walking in a structured community setting can improve function for older adults with functional limitations. In collaboration with others, recreation therapy (RT) and physical therapy (PT) students and faculty implemented the Walk On! program at a community church. Twenty-four African American females initially started the 11-week program and agreed to engage in the research aspect by completing pre/post-tests focused on physical function and quality of life, loneliness, and a geriatric depression scale. The hour-long biweekly intervention included warm up/cool down stretching, music, group motivation, walking around the large multipurpose room, and various balance activity stations interspersed. PT and RT students as well as faculty and community partners provided leadership and encouragement. Results were analyzed for 14 participants who completed all tests, as well as 75% of sessions. Data for the Geriatric Depression Scale and UCLA Loneliness Scale were analyzed using the Wilcoxon Signed Rank test. No statistical differences between pre/post-test scores were found. Visual inspection of GDS scores showed four participant scores increased slightly, meaning reporting more elements of depression, and one score decreased by 50%, indicating a drop from depression to not an issue. No changes were found in the other nine scores. The Loneliness Scale had slight changes for five participants with two increasing and three decreasing slightly. On the SF-36 Health Survey, Vitality increased or fatigue decreased (p=0.009) and Mental health or wellbeing increased (p=0.041). Further research on the psychosocial aspects of the Walk On! program in other populations will be conducted.

